# Deep learning-based clinical-radiomics nomogram for preoperative prediction of lymph node metastasis in patients with rectal cancer: a two-center study

**DOI:** 10.3389/fmed.2023.1276672

**Published:** 2023-12-01

**Authors:** Shiyu Ma, Haidi Lu, Guodong Jing, Zhihui Li, Qianwen Zhang, Xiaolu Ma, Fangying Chen, Chengwei Shao, Yong Lu, Hao Wang, Fu Shen

**Affiliations:** ^1^Department of Radiology, Changhai Hospital, The Navy Medical University, Shanghai, China; ^2^Department of Radiology, Ruijin Hospital, Shanghai Jiaotong University School of Medicine, Shanghai, China; ^3^Department of Colorectal Surgery, Changhai Hospital, The Navy Medical University, Shanghai, China

**Keywords:** rectal cancer, radiomics, magnetic resonance imaging, lymph node metastasis, deep learning

## Abstract

**Background:**

Precise preoperative evaluation of lymph node metastasis (LNM) is crucial for ensuring effective treatment for rectal cancer (RC). This research aims to develop a clinical-radiomics nomogram based on deep learning techniques, preoperative magnetic resonance imaging (MRI) and clinical characteristics, enabling the accurate prediction of LNM in RC.

**Materials and methods:**

Between January 2017 and May 2023, a total of 519 rectal cancer cases confirmed by pathological examination were retrospectively recruited from two tertiary hospitals. A total of 253 consecutive individuals were selected from Center I to create an automated MRI segmentation technique utilizing deep learning algorithms. The performance of the model was evaluated using the dice similarity coefficient (DSC), the 95th percentile Hausdorff distance (HD95), and the average surface distance (ASD). Subsequently, two external validation cohorts were established: one comprising 178 patients from center I (EVC1) and another consisting of 88 patients from center II (EVC2). The automatic segmentation provided radiomics features, which were then used to create a Radscore. A predictive nomogram integrating the Radscore and clinical parameters was constructed using multivariate logistic regression. Receiver operating characteristic (ROC) curve analysis and decision curve analysis (DCA) were employed to evaluate the discrimination capabilities of the Radscore, nomogram, and subjective evaluation model, respectively.

**Results:**

The mean DSC, HD95 and ASD were 0.857 ± 0.041, 2.186 ± 0.956, and 0.562 ± 0.194 mm, respectively. The nomogram, which incorporates MR T-stage, CEA, CA19-9, and Radscore, exhibited a higher area under the ROC curve (AUC) compared to the Radscore and subjective evaluation in the training set (0.921 vs. 0.903 vs. 0.662). Similarly, in both external validation sets, the nomogram demonstrated a higher AUC than the Radscore and subjective evaluation (0.908 vs. 0.735 vs. 0.640, and 0.884 vs. 0.802 vs. 0.734).

**Conclusion:**

The application of the deep learning method enables efficient automatic segmentation. The clinical-radiomics nomogram, utilizing preoperative MRI and automatic segmentation, proves to be an accurate method for assessing LNM in RC. This approach has the potential to enhance clinical decision-making and improve patient care.

**Research registration unique identifying number (UIN):**

Research registry, identifier 9158, https://www.researchregistry.com/browse-the-registry#home/registrationdetails/648e813efffa4e0028022796/.

## Introduction

Rectal cancer (RC) is a prevalent tumor affecting the gastrointestinal system and poses a significant global burden (1). The presence of lymph node metastasis (LNM) in RC individuals, particularly in cases defined as locally advanced rectal cancer (LARC), is associated with a poor prognosis. In order to manage LARC, the customary clinical strategy comprises administering neoadjuvant chemoradiotherapy (nCRT) before conducting total mesorectal excision (TME) surgery (2). This approach proves effective in diminishing the likelihood of local recurrence or the spread of cancer to distant sites (3). Achieving precision treatment in RC relies on accurate preoperative assessment of LNM (4). Consequently, it becomes crucial to accurately detect of lymph node (LN) involvement before surgery (4–6).

High-resolution magnetic resonance imaging (MRI) holds significant importance in the initial assessment of RC conditions. Nonetheless, achieving a precise preoperative diagnosis of LN involvement remains challenging in clinical practice (5). Relying solely on size as the exclusive criterion provides only acceptable precision. For instance, just 94% of the impacted LN possess a dimension less than 5 mm (6). A large node could be a successful tool to examine dimensions, perimeter, and signal intensity in LN. However, morphological criteria did not enhance the precision of lymph node staging in cases of RC (7). This challenge is further complicated by the absence of agreement regarding the relevant standards for evaluating LN contribution (7–9). Therefore, it is imperative to establish advanced and highly sensitive diagnostic tools to enhance the accuracy of LNM diagnosis in patients with RC.

Recently, several studies have demonstrated that radiomics can assist researchers in tackling diverse clinical tasks. By extracting numerous quantitative features from medical images through high-throughput analysis, radiomics approaches have the potential to empower radiologists to enhance diagnostic accuracy, ultimately benefiting patients (10–16). Radiomics-based models have exhibited promising value in detecting LNM in digestive tumors (10–12, 17–20). However, most existing methodologies rely on manual volume measurements of the entire primary tumor, which can be highly laborious, time-consuming, and subject to operator variability (12, 19, 20).

To the best of our knowledge, there is a lack of clear exploration regarding a deep learning-based image segmentation and clinical-radiomics nomogram for detecting LNM in individuals with RC. Therefore, the objective of this research was to create and validate an MR-based clinical-radiomics nomogram model that utilizes deep learning-based image segmentation. The purpose was to enable preoperative assessment of LNM and assess its clinical applicability in the context of RC.

## Materials and methods

### Participants

The trial followed the Declaration of Helsinki and had permission from the Ethics Committees of Changhai Hospital and Ruijin Hospital Luwan Branch. Written informed consent was waived as the retrospective design.

From January 2017 to January 2020, a total of 392 consecutive patients with RC diagnosed pathologically at Changhai Hospital (center I) were included in this retrospective trial. The inclusion criteria comprised the following: (1) histological diagnosis of rectal adenocarcinoma based on postoperative pathological examination; (2) presence of a single tumor focus; (3) baseline rectal magnetic resonance imaging (MRI) performed within 14 days prior to surgical resection. Exclusion criteria were as follows: (1) receipt of any local or systemic treatment prior to surgical resection (*n* = 86); (2) previous or concurrent diagnosis of cancers other than RC (*n* = 8); (3) poor image quality (*n* = 11); (4) synchronous distant metastasis (*n* = 22); (5) positive CRM (*n* = 7); (6) history of previous pelvic surgery (*n* = 5). Ultimately, a total of 253 cases were enrolled from center I. Additionally, another 178 patients from Changhai Hospital (temporal external validation center I, EVC1) and 88 patients from Ruijin Hospital Luwan Branch (spatial external validation center II, EVC2), who met the same exclusion criteria as external validation sets 1 and 2, were also included between February 2020 and May 2023 for external validation.

### Clinicopathologic data

Patient information and clinicopathologic findings were retrospectively obtained from the clinicopathological databases. This included data such as sex, age, BMI, histological differentiation, carbohydrate antigen 19-9 (CA19-9), carcinoembryonic antigen (CEA), circumferential resection margin (CRM), and pathological T-stage and N-stage. The CEA level was considered negative if it was less than 5 ng/mL, while the CA19-9 level was considered negative if it was less than 37 U/mL. These measurements were recorded at the same time as the baseline MRI. During the surgical procedure, all LN within the mesorectum were obtained from the surgical samples, ensuring a minimum of 12 lymph nodes were extracted per subject. The patients were categorized into different groups based on the National Comprehensive Cancer Network (NCCN) and American Joint Committee on Cancer (AJCC) staging system (21). The N0 group consisted of patients without lymph node metastasis (LNM), while the N1–2 group included patients with LNM.

### Image acquisition and analysis

Rectal MRI scans were conducted using either a 1.5 or 3.0 T MR systems (Siemens 1.5, 3.0, and GE 3.0 T) along with a phased array coil. Prior to the scan, a 20 mL glycerin enema was administered to perform intestinal cleansing. The standard imaging protocol included axial diffusion-weighted imaging (DWI) with a b-value of 0 and 1,000 s/mm^2^, sagittal T2-weighted imaging (T2WI), axial T1-weighted imaging (T1WI), and gadolinium contrast-enhanced T1WI of the pelvis in sagittal, coronal, and axial planes. Additionally, oblique axial high-resolution T2WI (HR-T2WI) images, which were perpendicular to the long axis of the rectum and included the lesion, were obtained. Supplementary Table 1 provides detailed information on the parameters used for HR-T2WI, which were utilized for the radiomics models.

Subjective evaluation of RC using MR imaging was conducted by three trained radiologists, namely R1, R2, and R3, with 12, 9, and 6 years of expertise, correspondingly. These radiologists were unaware of the pathological data. The assessment encompassed the evaluation of the subsequent tumor attributes: (1) tumor height, described as the measurement from the lower border of the tumor to the anal verge on MRI; (2) MR-reported T stage; (3) MR-reported N stage, and LN metastasis was identified if any of the following criteria was met: LN short-axis diameter superior to 10 mm, internal necrosis, nonuniform signal, LN fusion, nonuniform enhancement, or ill-defined borders (22, 23); (4) involvement of the mesorectal fascia (MRF); (5) presence of extramural venous invasion (EMVI). Any discrepancies among the radiologists’ evaluations were resolved through discussion until a consensus was reached by at least two of the experts. The interobserver correlation of subjective evaluation for LN metastasis between any two radiologists was assessed using the Kappa statistic. The intraclass correlation coefficient (ICC) was calculated to evaluate the consistency of subjective evaluation for LN metastasis among all three radiologists.

### Deep learning-based image segmentation

Since MR scans were performed using different MR scanners, the acquired DICOM data (oblique axial HR-T2WI) underwent preprocessing in these two centers. We adopted the data preprocessing strategy through data fingerprint information, including resampling strategy, cropping area size, gray value distribution, etc. information, thus forming a so-called “configuration plan.” The size of each raw image was first adjusted by cropping to a size of 384 × 384 × 64. Subsequently, all images were resampled to a target spacing of [0.36, 0.36, 0.36] mm to ensure a consistent target spacing. The preprocessed images were subsequently brought into ITK-SNAP software version 4.0.0^1^ for manual layer-by-layer segmentation of the entire RC lesion. This segmentation process aimed to obtain the volume of interest (VOI) representing the most accurate boundary fitting the primary tumor’s area for each case. These segmented images served as mask images (ground truth, GT) for the training of the segmentation neural network.

The initial cohort of 253 cases from center I was randomly split into a network training set (60%, *n* = 152) and a network test set (40%, *n* = 101) for the development and validation of an automated segmentation method using nnU-Net during Stage I of our research. nnU-Net is a self-configuring approach specifically designed for deep learning-based segmentation of biomedical images (24). The details of the segmentation neural network can be found in Supplementary Figure 1. To mitigate overfitting, we implemented data augmentation along with 5-fold cross-validation. Additionally, the dice similarity coefficient (DSC), the 95th percentile Hausdorff distance (HD95), and average surface distance (ASD) between the automatically segmented images and the GT images were also reported in Supplementary Figure 2.

Then, the tested cases for automatic segmentation (*n* = 101) were also employed as a subsequent training set for the model to facilitate LNM classification in Stage II, thus avoiding excessively time-consuming processes. As for the segmentation task in Stage II, we also learned from the “configuration plan” and selected a parameter setting with a centered distribution. The automatic segmentation process was repeated with a one-week time interval to assess feature consistency. Finally, the Artificial Intelligence Kit software (GE Healthcare) was utilized to extract features from all automatically delineated VOIs derived from the model training set (n = 101), EVC1 (*n* = 178), and EVC2 (*n* = 88).

### Radiomics feature extraction and reduction

Based on the automatically delineated VOIs, four categories of features were identified. These included: (1) first-order features, which describe the voxel intensity distribution on MR images, (2) shape features, which capture the 3D properties of the VOIs, (3) texture features, which quantify the dissimilarities in heterogeneity within the region using techniques such as size zone, run length, gray-level co-occurrence, and neighborhood gray-tone difference matrices, and (4) higher-order features, which are derived from transformed first-order data and texture features. This category includes square, square root, logarithm, exponential, gradient, local binary pattern (LBP), and wavelet transformations.

The intraclass correlation coefficient (ICC) was calculated to evaluate the robustness of the features during model training. Only indexes with an ICC value above 0.8 were considered for further analysis. To identify the most relevant features associated with LNM, the Select K Best method and the least absolute shrinkage and selection operator (LASSO) algorithm were employed to develop a Radscore. The detailed process of feature selection can be found in Supplementary Figure 3.

### Nomogram model building and validation

**Figure 1:**
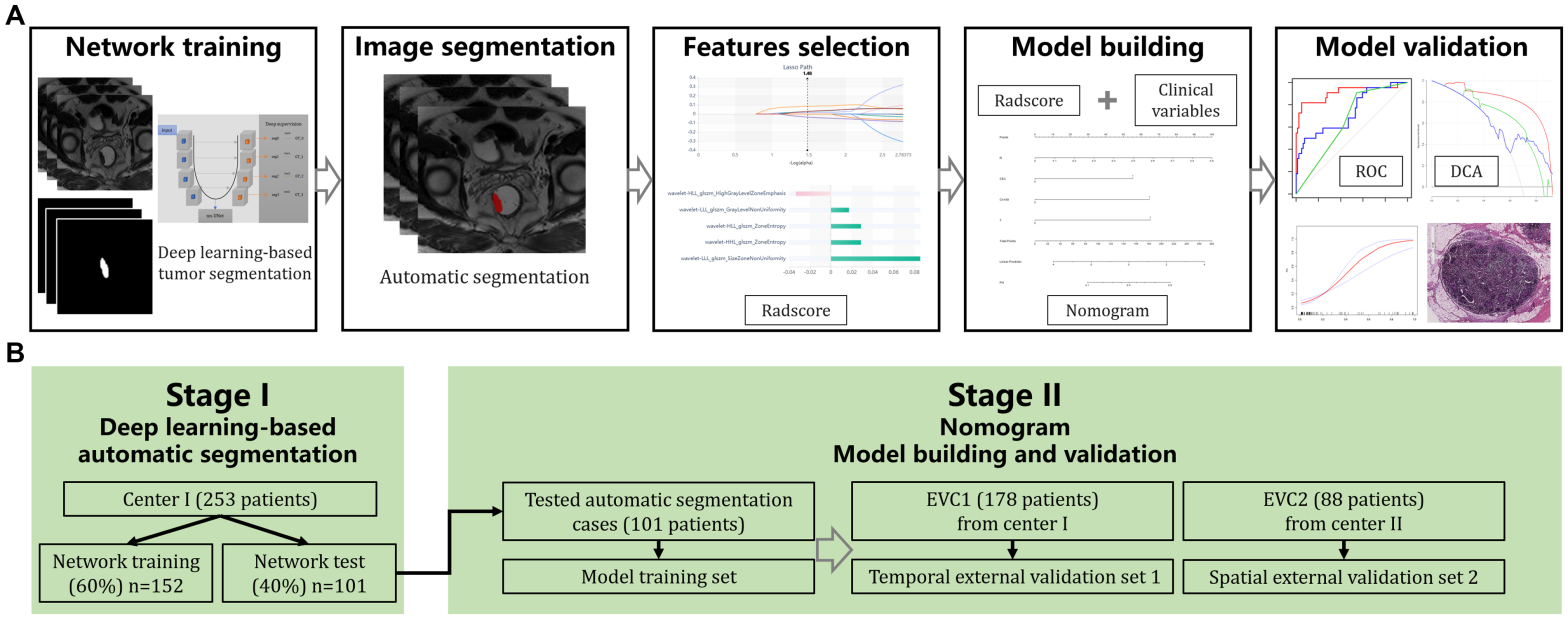
Study flowchart **(A)** and modeling methods **(B)**. Center 1, Changhai Hospital; EVC1, temporal external validation center I, Changhai Hospital; EVC2, spatial external validation center II, Ruijin Hospital Luwan Branch.

The predictive value of clinical features and the Radscore in detecting LNM was assessed through univariable logistic regression evaluation in the model training set. Factors with p lower than 0.05 were then used to develop a nomogram model through multiple factor logistic regression. Receiver operating characteristic (ROC) curve analysis was conducted to evaluate the performance of the Radscore, nomogram, and subjective evaluation model. External validation sets 1 and 2 were used to validate the accuracy of the detection (25). The models were compared using the DeLong test, and the goodness-of-fit of the nomogram was determined employing the Hosmer-Lemeshow test and calibration curves. To assess the comprehensive benefits, decision curve analysis (DCA) was employed. The study’s workflow is depicted in Figure 1.

### Statistical analysis

Statistical analysis was conducted using SPSS software (v. 26.0, IBM) and R package (v. 3.5.1, http://www.Rproject.org). Categorical data were analyzed using the Pearson chi-square test or Fisher’s exact test, whereas continuous data (mean ± standard deviation) were assessed using the Student’s t-test or Mann–Whitney *U*-test. A significance level of <0.05 (two-sided) was used to determine statistical significance.

## Results

### Patient features

The three cohorts exhibited no significant variations in demographic characteristics (all *p* > 0.05), as indicated in Table 1. Based on the pathological reports, LNM was identified in 50 out of 253 cases (19.8%) in center I, compared to 36 out of 178 cases (20.2%) in EVC1 and 24 out of 88 cases (27.3%) in EVC2. The interobserver agreement for the subjective evaluation of MR N-stage across all cohorts is presented in Supplementary Table 2.

**Table 1:** Patient demographics.

Variables		Center I	EVC1	EVC2	*p*-value
		*n* = 253	*n* = 178	*n* = 88	
Gender					0.849
	Male	173 (68.4%)	124 (69.7%)	63 (71.6%)	
	Female	80 (31.6%)	54 (30.3%)	25 (28.4%)	
Age (years)		58.420 ± 12.112	56.750 ± 11.357	57.830 ± 10.254	0.442
BMI (kg/m^2^)		23.434 ± 2.944	24.148 ± 2.968	23.334 ± 2.611	1.000
Tumor height (cm)^*^		4.751 ± 2.043	3.813 ± 1.864	4.773 ± 1.987	0.926
Pathological T-stage					0.187
	T1–2	117 (46.2%)	74 (41.6%)	47 (53.4%)	
	T3–4	136 (53.8%)	104 (58.4%)	41 (46.6%)	
Pathological N-stage					0.308
	N0	203 (80.2%)	142 (79.8%)	64 (72.7%)	
	N1–2	50 (19.8%)	36 (20.2%)	24 (27.3%)	
Differentiation					0.145
	High-moderate	200 (79.1%)	153 (86.0%)	69 (78.4%)	
	Poor	53 (20.9%)	25 (14.0%)	19 (21.6%)	
MR T-stage					0.366
	T1–2	148 (58.5%)	116 (65.2%)	55 (62.5%)	
	T3–4	105 (41.5%)	62 (34.8%)	33 (37.5%)	
MR N-stage					0.069
	N0	120 (47.4%)	67 (37.6%)	44 (50.0%)	
	N1–2	133 (52.6%)	111 (62.4%)	44 (50.0%)	
MRF					0.334
	Negative	202 (79.8%)	152 (85.4%)	72 (81.8%)	
	Positive	51 (20.2%)	26 (14.6%)	16 (18.2%)	
EMVI					0.696
	Negative	162 (64.0%)	121 (68.0%)	58 (65.9%)	
	Positive	91 (36.0%)	57 (32.0%)	30 (34.1%)	
CEA^**^					0.844
	Negative	165 (65.2%)	112 (62.9%)	55 (62.5%)	
	Positive	88 (34.8%)	66 (37.1%)	33 (37.5%)	
CA19-9^**^					0.139
	Negative	204 (80.6%)	130 (73.0%)	71 (80.7%)	
	Positive	49 (19.4%)	48 (27.0%)	17 (19.3%)	

BMI, body mass index; CEA, carcinoembryonic antigen; CA19-9, carbohydrate antigen 19-9; MRF, mesorectal fascia; EMVI, extramural venous invasion. ^*^Tumor height was defined as the distance between the lower edge of the tumor and the anal verge by MRI. ^**^Preoperative blood samples at the same time as baseline MRI. Center I, Changhai Hospital; EVC1, external validation from center I, Changhai Hospital; EVC2, external validation from center II, Ruijin Hospital Luwan Branch.

### Automatic segmentation results

**Figure 2:**
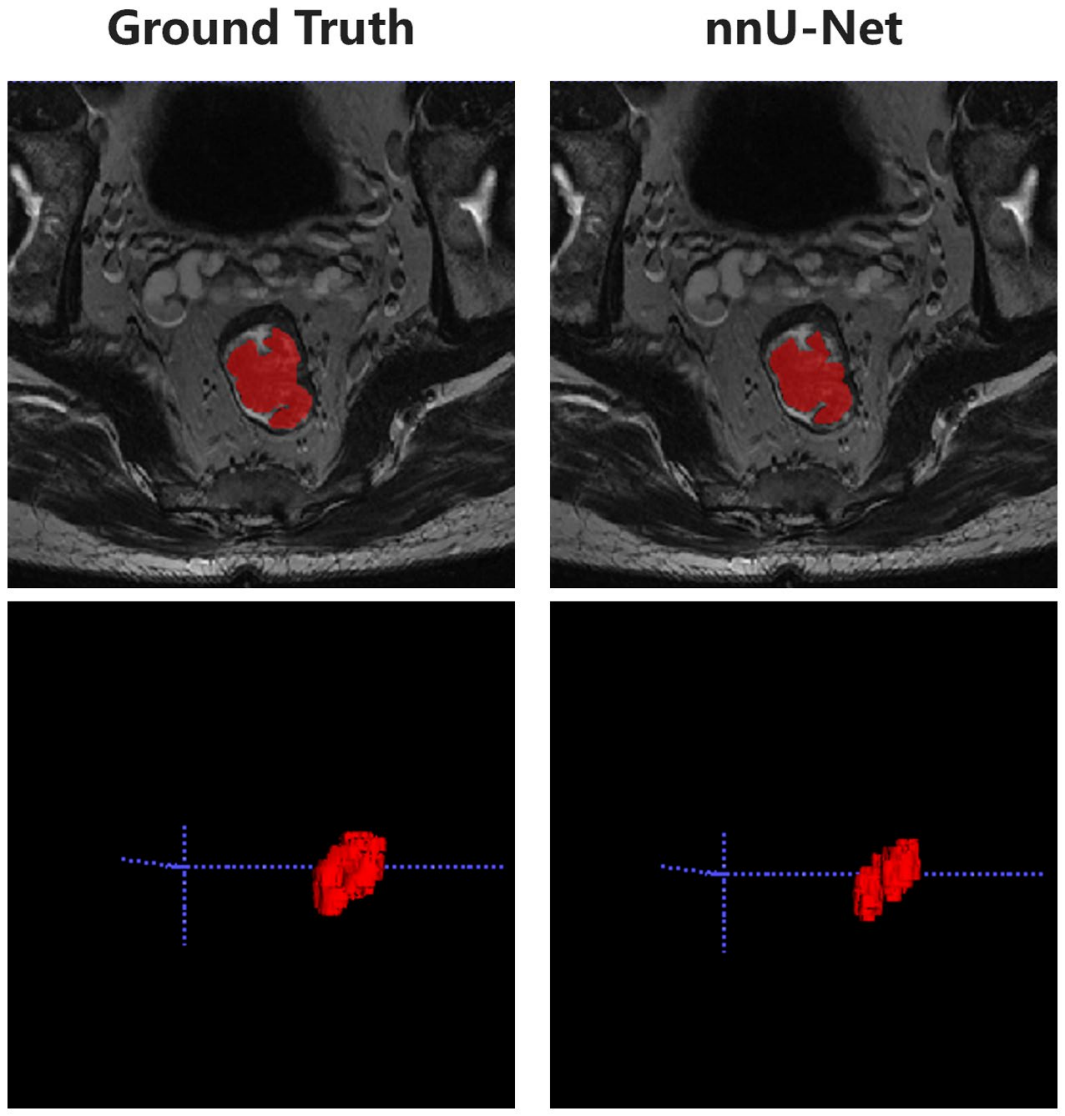
Representative diagram of automatic segmentation.

The developed deep learning-based automatic segmentation method demonstrates the capability to execute automated configuration for our datasets, effectively encompassing the entire lesion in HR-T2WI (Figure 2). The mean DSC, HD95, and ASD between the automatic segmentation and GT were 0.857 ± 0.041, 2.186 ± 0.956 mm, and 0.562 ± 0.194 mm, respectively (Supplementary Figure 2).

### Model building and evaluation

**Figure 3:**
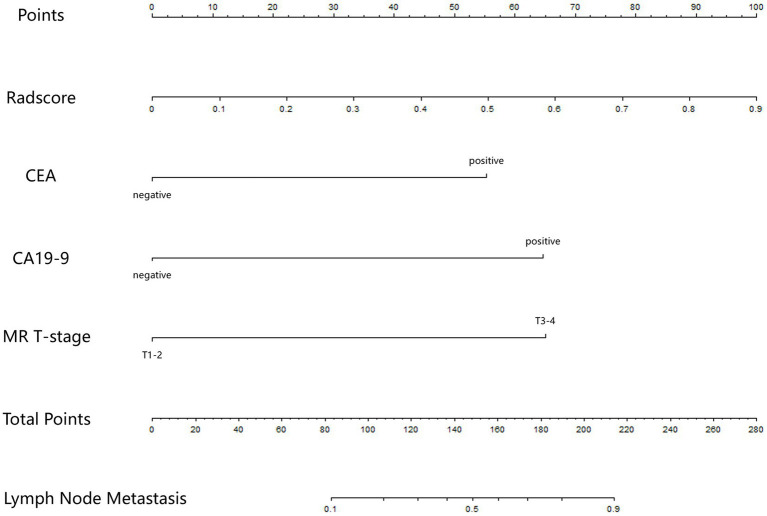
The nomogram. In the visual nomogram, first, a vertical line was drawn according to the values of the most influential factors to determine the corresponding numbers of points. The total points were the sum of the above points. Then, a vertical line was drawn according to the value of total points to determine the probability of LNM.

**Figure 4:**
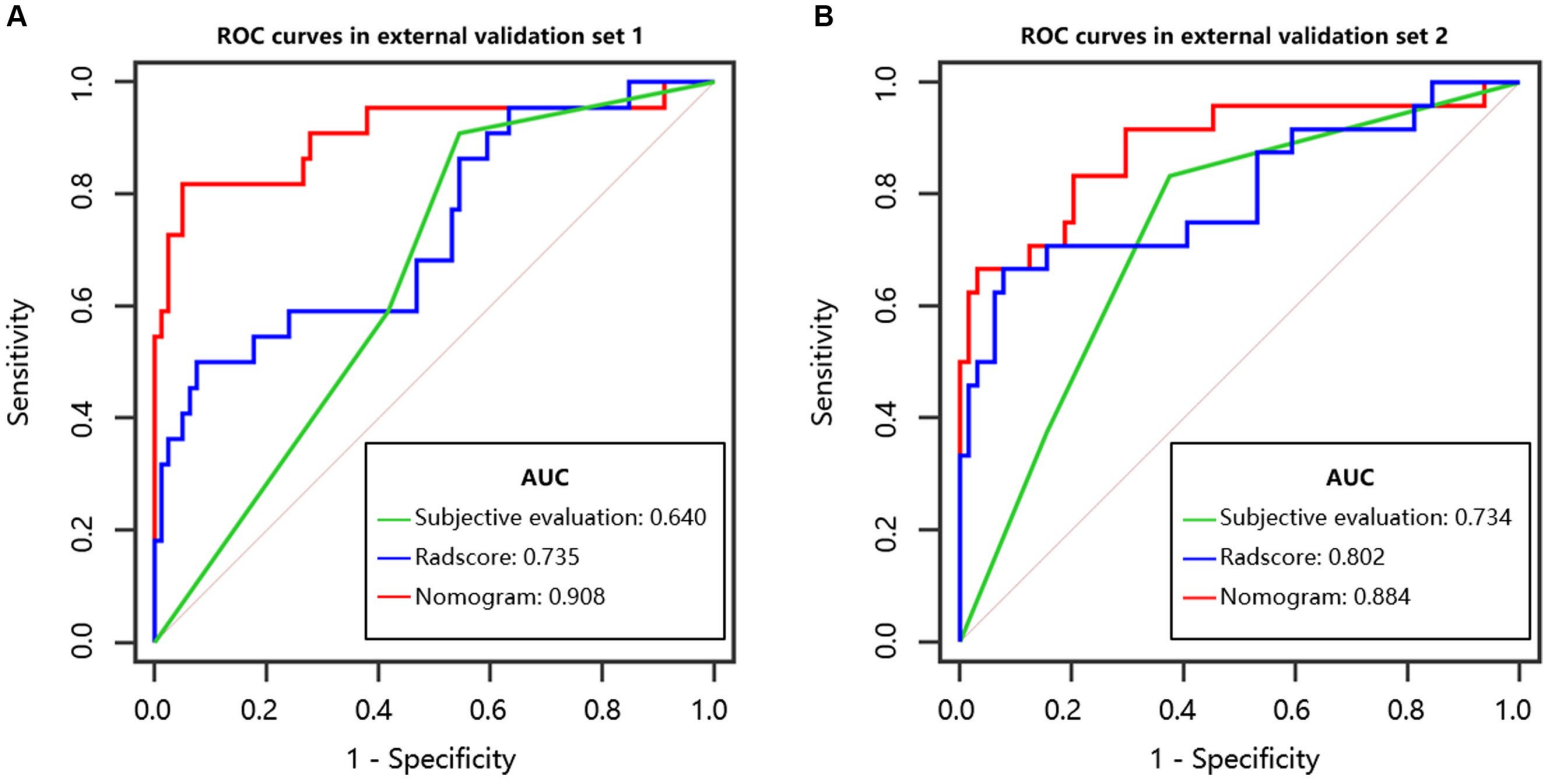
ROC curves. **(A)** External validation set 1. **(B)** External validation set 2.

**Figure 5:**
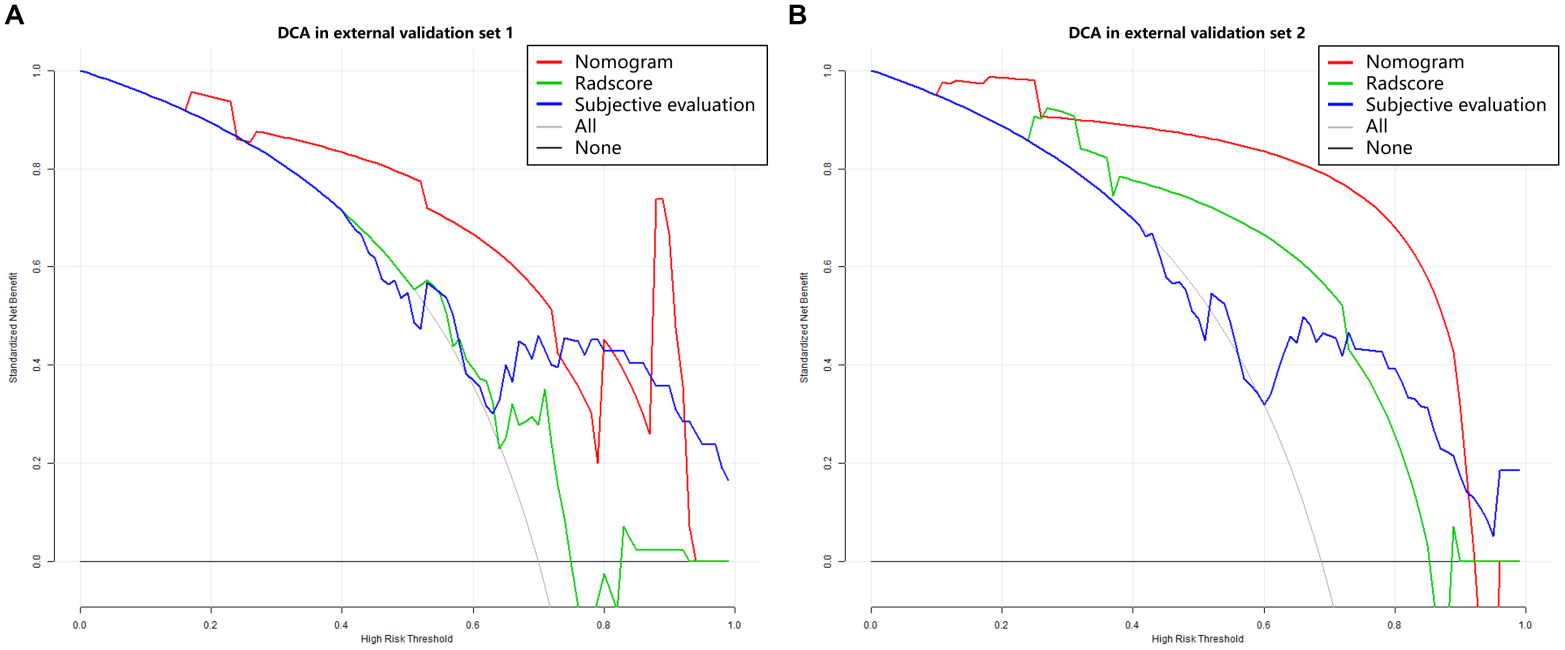
Decision curve analysis. **(A)** External validation set 1. **(B)** External validation set 2. The Y-axis represents the net benefit, calculated by adding true positives and subtracting false positives. The X-axis corresponds to the probability threshold (depicted as a solid line with a scale at the bottom). The light and dark gray lines indicate assumptions that all cases or no cases have lymph node metastasis (referred to as the “all” or “none” scheme), respectively. Red, green and blue curves showed that with a large probability range, utilizing the developed nomogram to predict the odds of LNM conferred a positive net benefit vs. the Radscore, subjective evaluation and the all-or-none scheme.

In the model training set, five features were identified and utilized to develop a Radscore, as shown in Table 2 and Supplementary Figure 3. Univariable analysis demonstrated a significant association between LNM and the following factors: MR T-stage, MR N-stage, CEA, CA19-9, and Radscore (Table 3). Subsequently, a nomogram model was constructed using multivariable logistic regression analysis, considering the selected risk factors (MR T-stage, CEA, CA19-9, and Radscore, as indicated in Table 4). The probabilities were calculated using the formula: −4.97107 + 3.72165 * Radscore + 1.85358 * CEA + 2.16416 * CA199 + 2.18032 * MR T-stage, resulting in an AUC of 0.921 (Supplementary Table 3). The generated nomogram, presented in Figure 3, exhibited a higher AUC compared to the Radscore and subjective evaluation in both external validation sets (0.908 vs. 0.735 vs. 0.640, and 0.884 vs. 0.802 vs. 0.734). These statistically significant differences were confirmed by the DeLong test. Detailed ROC analyses can be found in Table 5 and Figure 4. Calibration curves for the nomogram in both validation datasets indicated no significant deviation (Hosmer-Lemeshow test, *p* = 0.065 and 0.610) from an ideal fit (Supplementary Figure 4). DCA demonstrated that utilizing the nomogram model to assess the probability of LNM offered a positive net benefit compared to the Radscore, subjective evaluation, and the all-or-none approach at a significant threshold probability (Figure 5).

**Table 2:** Description of the selected radiomics features.

Radiomics feature	Radiomics class	Filter
Size zone non-uniformity	GLSZM	wavelet-LLL
Gray level non-uniformity	GLSZM	wavelet-LLL
Zone entropy	GLSZM	wavelet-HLL
High gray level zone emphasis	GLSZM	wavelet-HLL
Zone entropy	GLSZM	wavelet-HHL

GLSZM, Gray-level size zone matrix.

**Table 3:** Univariate analysis in training set.

Variables	Pathological N stage			Univariate logistic regression	
	Total (*n* = 101)	N0 (*n* = 79)	N1–2 (*n* = 22)	OR (95% CI)	*p*-value
Gender					0.897
Male	77 (76.2%)	60 (76.0%)	17 (77.3%)	1.0 (reference)	
Female	24 (23.8%)	19 (24.0%)	5 (22.7%)	0.929 (0.302, 2.854)	
Age (years)	56.139 ± 11.335	56.671 ± 10.789	54.227 ± 13.212	0.982 (0.942, 1.022)	0.371
BMI (kg/m^2^)	23.402 ± 2.947	23.545 ± 2.904	22.888 ± 3.110	0.925 (0.785, 1.090)	0.355
Tumor height (cm)	4.743 ± 2.057	4.785 ± 2.170	4.591 ± 1.623	0.953 (0.751, 1.210)	0.695
MR T-stage					**<0.001**
T1–2	66 (65.3%)	60 (76.0%)	6 (27.3%)	1.0 (reference)	
T3–4	35 (34.7%)	19 (24.0%)	16 (72.7%)	8.421 (2.886, 24.570)	
MR N-stage					**0.030**
N0	39 (38.6%)	36 (45.6%)	3 (13.6%)	1.0 (reference)	
N1–2	62 (61.4%)	43 (54.4%)	19 (86.4%)	1.887 (1.063, 3.351)	
MRF					0.116
Negative	81 (80.2%)	66 (83.5%)	15 (68.2%)	1.0 (reference)	
Positive	20 (19.8%)	13 (16.5%)	7 (31.8%)	2.369 (0.807, 6.952)	
EMVI					0.208
Negative	65 (64.4%)	54 (68.4%)	11 (50.0%)	1.0 (reference)	0.116
Positive	36 (35.6%)	25 (31.6%)	11 (50.0%)	2.160 (0.826, 5.646)	
CEA					**<0.001**
Negative	66 (65.3%)	61 (77.2%)	5 (22.7%)	1.0 (reference)	
Positive	35 (34.7%)	18 (22.8%)	17 (77.3%)	11.522 (3.732, 35.571)	
CA19-9					**<0.001**
Negative	82 (81.2%)	72 (91.1%)	10 (45.4%)	1.0 (reference)	
Positive	19 (18.8%)	7 (8.9%)	12 (54.6%)	12.343 (3.936, 38.709)	
Radscore	0.263 ± 0.203	0.219 ± 0.153	0.421 ± 0.275	84.761 (8.301, 865.471)	**<0.001**

LNM, lymph node metastasis; OR, odds ratio. The meaning of bold values provided in table was *p*-value < 0.05.

**Table 4:** Multivariate analysis in training set.

Variables		Training set (*n* = 101)	
		OR (95% CI)	*p*-value
MR T-stage			**0.005**
	T1–2	1.0 (reference)	
	T3–4	9.344 (1.973, 44.259)	
MR N-stage			0.050
	N0	1.0 (reference)	
	N1–2	2.513 (1.000, 6.317)	
CEA			**0.010**
	Negative	1.0 (reference)	
	Positive	7.810 (1.6252, 37.5291)	
CA19-9			**0.011**
	Negative	1.0 (reference)	
	Positive	9.418 (1.654, 53.641)	
Radscore		39.242 (1.540, 999.808)	**0.026**

LNM, lymph node metastasis; OR, odds ratio. The meaning of bold values provided in table was *p*-value < 0.05.

**Table 5:** ROC analysis in validation sets.

	External validation set 1			External validation set 2		
	Subjective evaluation	Radscore	Nomogram	Subjective evaluation	Radscore	Nomogram
AUC	0.640	0.735	0.908	0.734	0.802	0.884
95% CI	0.539 to 0.733	0.638 to 0.818	0.834 to 0.956	0.629 to 0.823	0.704 to 0.879	0.798 to 0.943
Sensitivity	86.4%	50.0%	81.8%	83.3%	66.7%	66.7%
Specificity	45.6%	92.4%	94.9%	62.5%	92.2%	96.9%
Accuracy	54.5%	83.2%	92.1%	71.6%	85.2%	88.6%
PLR	1.670	6.583	16.159	2.222	8.533	21.333
NLR	0.199	0.541	0.191	0.267	0.362	0.344
PPV	0.317	0.647	0.818	0.455	0.762	0.889
NPV	0.947	0.869	0.949	0.909	0.881	0.886
*p-*value^*^	<0.001	<0.001		0.018	0.035	

^*^Compared with nomogram by DeLong test. AUC, area under the curve; PLR, positive likelihood ratio; NLR, negative likelihood ratio; NPV, negative predictive value; PPV, positive predictive value.

## Discussion

Here, we focused on the development and validation of a deep learning-based image segmentation method for accurate delineation of rectal adenocarcinoma. Subsequently, a clinical-radiomics nomogram was constructed, demonstrating significantly improved performance compared to the Radscore and subjective evaluation when assessing lymph node metastasis (LNM) in patients with rectal cancer (RC). Radiologists and clinicians can utilize this intelligent, noninvasive, intuitive, and convenient approach to obtain personalized predictive information through straightforward calculations prior to surgery.

In patients with RC, preoperative detection of LNM plays a crucial role in tumor staging and treatment decision-making. It provides fundamental information for individualized treatment approaches, which primarily include surgical resection and nCRT, with variations based on the pathological stage of the lesion (2). Precise LN staging in RC is crucial to appropriately select individuals for preoperative procedure, ensuring avoidance of undertreatment and minimization of overtreatment. However, conventional magnetic resonance imaging (MRI) falls short in accurately detecting LN metastasis, exhibiting suboptimal sensitivity, accuracy, and specificity (7–9, 26). This suggests that subjective MRI standards for LNM detection are unreliable, primarily due to the absence of a consensus on appropriate morphological criteria for accurate assessment of LN involvement. The data from our validation sets confirmed that the subjective evaluation demonstrates acceptable sensitivity for detecting LNM, ranging from 86.4% to 83.3%. However, the specificity is relatively low, ranging from 45.6 to 62.5%, which aligns with our clinical experience. Meanwhile, the accuracies of subjective MR N-stage were 54.5% to 71.6%. This negative influence becomes more pronounced when constructing a clinical-radiomics nomogram, leading to the exclusion of subjective MR N-stage from the final nomogram model in current research.

Radiomics represents a novel approach that utilizes routine imaging findings to conduct high-throughput quantitative evaluations. This quantitative method offers a noninvasive tool for the detailed analysis of the biological properties and variability of RC, surpassing the limitations of morphological visual representation. Currently, several studies (10–12) have showcased the viability of radiomics in predicting LNM in CRC. Our previous study (12) developed a radiomics model for primary lesions in RC using a random forest (RF) classifier to LNM. The RF demonstrated an AUC of 0.746, serving as a performance evaluation of diagnostics. However, the sensitivity and specificity of the model still fell below 80%. One potential explanation for this is the absence of clinicopathological risk factors in the model.

It is worth noting that we developed a clinical-radiomics nomogram model that combines MR T-stage, CEA, CA19-9, and Radscore. This model serves as an intuitive visualization tool with enhanced discriminatory ability for preoperative detection of LNM. It demonstrated favorable performance and superior diagnostic efficiency compared to subjective evaluation (*p* < 0.05). Furthermore, our findings suggest that the combination of Radscore and clinical factors outperformed the radiomics signature alone in predicting LNM in rectal adenocarcinoma. The addition of clinical factors resulted in an elevated AUC (0.802 to 0.884), along with significantly higher specificity (96.9%) and PLR of 21.333 in the external validation cohort. Consequently, a preoperative nomogram which can be trained effectively and explained easily was developed to assist radiologists and clinicians in assessment of LNM intuitively and rapidly.

Moreover, this study utilized radiomics features extracted from automatic segmentation based on deep learning. Specifically, we employed 60% of the center 1 dataset for training a neural network called nnU-Net, which enables automated image segmentation in HR-T2WI. Although nnU-net is a unified framework, the original architecture displays strong generalization characteristics requiring neither expert knowledge nor compute resources beyond standard network training in various medical image segmentation challenges (24). Compared to the conventional manual approach, the automated image segmentation offers convenience, eliminates the risk of perceptual errors, and is well-suited for processing substantial amounts of records. As a standardized and dataset-agnostic framework, nnU-Net was proposed as a robust and powerful tool for medical image segmentation (24). This streamlined and efficient procedure has the potential to alleviate the burden of the often laborious and inconsistent manual segmentation process. By leveraging artificial intelligence, this approach enhances the reliability of research and holds promise as a replacement for the time-consuming and non-reproducible manual segmentation method currently in use (27).

The inclusion of two distinct validation cohorts from external sources was another noteworthy aspect of this research. Consistent with the findings in the training set, the temporal and spatial external validation cohorts exhibited favorable discrimination, calibration, and improved clinical utility when utilizing the nomogram. This suggests that incorporating an external dataset can help mitigate the limitations of overfitting associated with a novel model. Consequently, the nomogram model holds the potential to enhance diagnostic confidence for radiologists and offer clinicians a more valuable and objective understanding of overall prognostic factors prior to clinical decision-making.

This investigation had several limitations that should be acknowledged. Firstly, the sample size was small, and the study design was retrospective, which may introduce selection bias and limit the general applicability of the findings. Therefore, larger-scale multicenter studies are required to overcome these limitations and validate the results more robustly. Additionally, the imaging segmentation was conducted automatically based on the primary tumor in RC. While most methodologies emphasize the use of the entire tumor volume, this study only extracted and analyzed radiomics features from the primary tumor itself, without exploring the features of the LN. This limitation may lead to incomplete observation data and potentially impact the overall analysis. This point has garnered significant attention in both theoretical and application domains. However, deep learning approaches for the direct identification of LNM have not been developed and validated in this research. Multiple prior studies provide evidence that DL models can effectively predict tumor heterogeneity in rectal cancer, covering aspects like lymph node metastasis, distant metastasis, and patient survival (28–31). Nevertheless, deep learning investigations vary widely, and these models often lack interpretability. Although it is not easy for deep learning models to become explanatory and reasonable, which still puzzles many researchers. The application of artificial intelligence methods has the potential to guide personalized treatment plans, offering an emerging prognostic approach that warrants further investigation in the future (32–34).

## Conclusion

In summary, this study effectively developed and confirmed a clinical-radiomics nomogram by utilizing preoperative rectal MRI and automated segmentation. The nomogram incorporated both the Radscore and clinical risk factors, demonstrating its usefulness in predicting LNM. This innovative nomogram model demonstrated enhanced clinical utility compared to subjective evaluation and the Radscore alone. This noninvasive approach has the potential to intelligently enhance risk stratification in rectal cancer and can be readily applied in a clinical setting.

## Data availability statement

The raw data supporting the conclusions of this article will be made available by the authors, without undue reservation.

## Ethics statement

The studies involving humans were approved by the Ethics Committee of the Changhai Hospital, Naval Medical University. The studies were conducted in accordance with the local legislation and institutional requirements. The ethics committee/institutional review board waived the requirement of written informed consent for participation from the participants or the participants' legal guardians/next of kin because written informed consent was waived as the retrospective design. Written informed consent was not obtained from the individual(s) for the publication of any potentially identifiable images or data included in this article because written informed consent was waived as the retrospective design.

## Author contributions

SM: Writing – original draft, Data curation, Formal Analysis, Investigation. HL: Writing – original draft, Methodology, Data curation. GJ: Writing – original draft, Data curation, Investigation. ZL: Data curation, Writing – original draft, Validation. QZ: Data curation, Writing – original draft. XM: Methodology, Data curation, Writing – original draft. FC: Data curation, Writing – original draft. CS: Funding acquisition, Supervision, Writing – review & editing. YL: Conceptualization, Funding acquisition, Methodology, Validation, Writing – review & editing. HW: Methodology, Conceptualization, Funding acquisition, Project administration, Resources, Writing – review & editing. FS: Conceptualization, Methodology, Writing – review & editing, Data curation, Funding acquisition, Project administration, Resources, Supervision.

## Footnotes

^1^ www.itksnap.org
