# Supplementary material for: Deep learning-based clinical-radiomics nomogram for preoperative prediction of lymph node metastasis in patients with rectal cancer: a two-center study
Source: Front Med (Lausanne). 2023 Dec 1;10:1276672. doi: 10.3389/fmed.2023.1276672 (PMC10722265; doi:10.3389/fmed.2023.1276672)
Supplement: Supplementary file 1 [file Data_Sheet_1.docx]

**Supplementary Materials**

**Supplemental Table 1. Oblique axial high-resolution T2WI sequence parameters**

| Cohort | Scanner | Field Strength | Sequence name | Echo train length | TR/TE (m/s) | Matrix | FOV  (mm) | Section thickness (mm) | FA (°) | TA |
| --- | --- | --- | --- | --- | --- | --- | --- | --- | --- | --- |
| CH | SIEMENS | 3.0 T | TSE | 16 | 4000/108 | 320×320 | 180×180 | 3 | 160 | 4 min 10 sec |
|  | GE | 3.0 T | FSE | 32 | 7845/109 | 352×352 | 200×200 | 4 | 110 | 2 min 11sec |
| RJ | SIEMENS | 1.5 T | FSE | 15 | 4000/90 | 320×320 | 180×180 | 3 | 150 | 4 min 08 sec |

FA: Flip angle; FOV: Field of view; TA: Acquisition time; TR/TE: Repetition time/echo time; TSE: Turbo spin echo; FSE: Fast spin echo.

CH: Shanghai Changhai Hospital.

RJ: Ruijin Hospital Luwan Branch.

**Supplemental Table 2. Interobserver agreement for the subjective evaluation of MR N-stage in all cohorts**

| **Dataset** | **Radiologist 1 - Radiologist 2**  ***Kappa* value (95% CI)** | **Radiologist 1 - Radiologist 3**  ***Kappa* value (95% CI)** | **Radiologist 2 - Radiologist 3**  ***Kappa* value (95% CI)** | **All Radiologists**  **ICC (95% CI)** |
| --- | --- | --- | --- | --- |
| **Center I** | 0.728 (0.674 - 0.803) | 0.647 (0.554 - 0.707) | 0.719 (0.694 - 0.842) | 0.773 (0.712 - 0.804) |
| **EVC1** | 0.842 (0.748 - 0.936) | 0.537 (0.385 - 0.689) | 0.650 (0.514 - 0.786) | 0.696 (0.631 - 0.755) |
| **EVC2** | 0.731 (0.677 - 0.775) | 0.681 (0.656 – 0.743) | 0.722 (0.677 - 0.859) | 0.718 (0.660 - 0.782) |

ICC: intraclass correlation coefficient

**Supplemental Table 3. ROC analysis in training set**

|  | **Subjective evaluation** | **Radscore** | **Nomogram** |
| --- | --- | --- | --- |
| **AUC** | 0.662 | 0.903 | 0.921 |
| **95% CI** | 0.587 to 0.731 | 0.850 to 0.942 | 0.872 to 0.956 |
| **Specificity** | 45.4% | 90.9% | 93.9% |
| **Sensitivity** | 86.9% | 89.7% | 90.3% |
| **Accuracy** | 79.2% | 89.9% | 91.0% |
| **PPV** | 0.875 | 0.977 | 0.985 |
| **NPV** | 0.792 | 0.667 | 0.689 |
| **PLR** | 1.593 | 9.862 | 14.907 |
| **NLR** | 0.288 | 0.114 | 0.103 |
| ***P* value ^*^** | <0.0001 | 0.311 |  |

^*^ Compared with nomogram by DeLong test

*AUC*: area under the curve; *PLR*: positive likelihood ratio; *NLR*: negative likelihood ratio; *NPV*: negative predictive value; *PPV*: positive predictive value.

**The details of data augmentation.**

Data augmentation is a strategy that mainly involves random transformation of the original data with a certain probability when the amount of data is small to prevent the model from overfitting. The data augmentation used in the experiment include random rotation, reduced scaling, contrast changes, resolution transformation, Gaussian noise, etc. The main purpose is to enhance the sample data in each batch of the training process. This means that the data augmentation for the data in the batch in each iteration and each epoch is different. Of course, there is a high probability that the original data will be maintained without data enhancement.

**Supplemental Figure 1.** **The automatic segmentation protocols.**


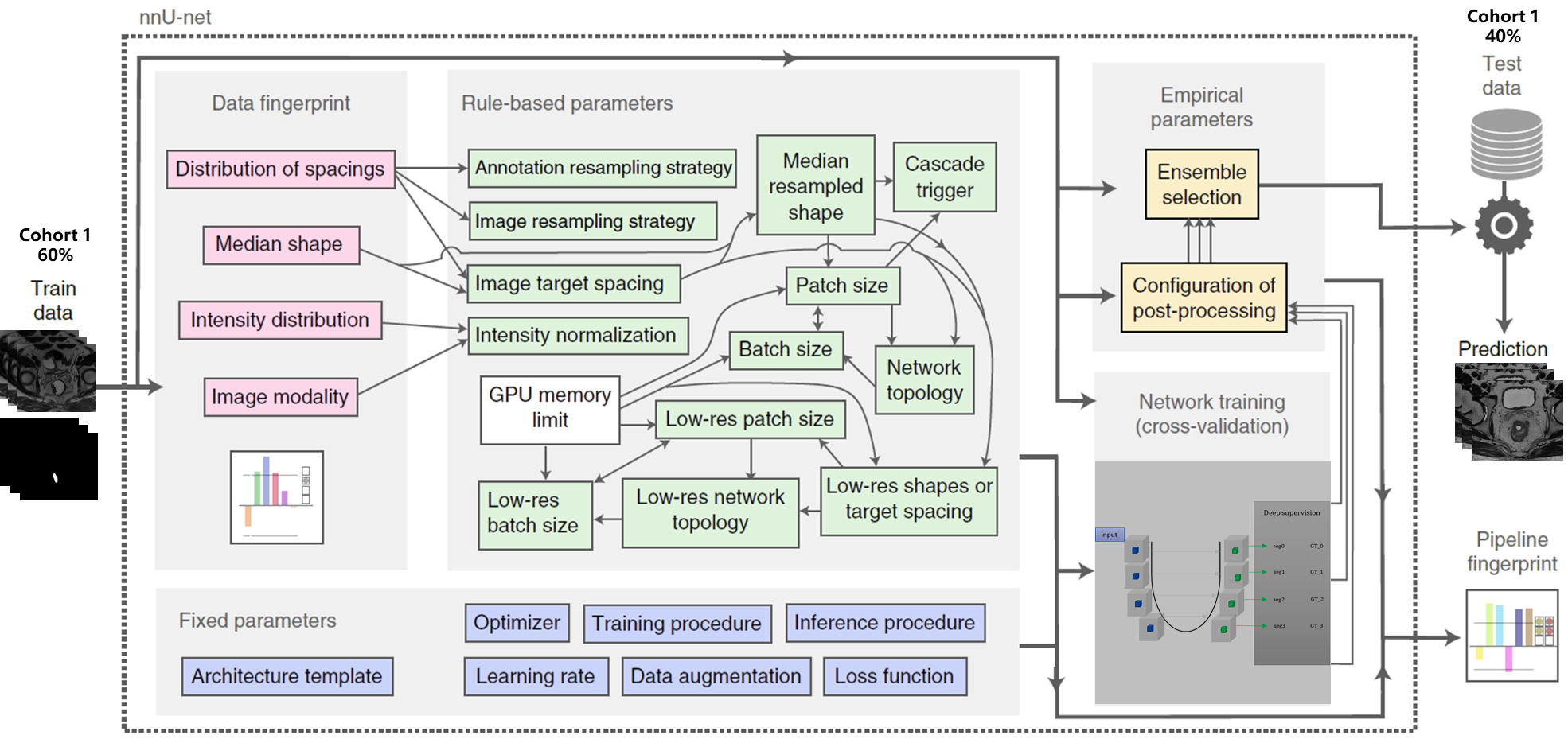


We implemented all the experiments based on Python 3.8, Pytorch 1.12.0, and Ubuntu 20.04. All training procedures have been run on a single NVIDIA TITAN RTX with 24GB memory. For the segmentation task, the initial learning rate was set to 0.01 with a “poly” decay strategy as described in Equation 1. The network parameters are optimized with Stochastic Gradient Descent (SGD) with a momentum of 0.99. The weight decay is set to 3e-5. The number of training epochs (i.e., max_epochs) is 1000 and contains 250 iterations every epoch.

$lr=initial_{lr}\times{(1-\frac{epoch}{max\_epoch})}^{0.9}$. (1)

We used a joint cross-entropy and soft dice loss to be the supervised loss function, which is given as in Equation 2:

$\mathcal{L}_{seg}\{h, w,d\}=\frac{1}{2}[\mathcal{L}_{CE}\left\{ h,w,d \right\}+\mathcal{L}_{Dice}\left\{ h,w,d \right\}]$. (2)

Cross Entropy Loss, which can be traced back to the information entropy proposed by Shannon, is a common basic classification loss function in machine learning. It is used to determine how close the actual output is to the desired output.

We also added deep supervision during the training stage. More specifically, the output of each stage in the decoder would be passed to the final expanding block, where cross-entropy loss and dice loss would be applied. Practically, we down-sampled the ground truth segmentation mask so as to match the resolution of the model’s prediction. Therefore, the final training objective function is the sum of all losses at four resolutions in Equation 3.

$\mathcal{L}_{all}=\delta_{1}\mathcal{L}_{seg}\left\{ h,w,d \right\}+\delta_{2}\mathcal{L}_{seg}\left\{ \frac{h}{4},\frac{w}{4},\frac{d}{2} \right\}+\delta_{3}\mathcal{L}_{seg}\left\{ \frac{h}{8},\frac{w}{8},\frac{d}{4} \right\}+\delta_{4}\mathcal{L}_{seg}\left\{ \frac{h}{16},\frac{w}{16},\frac{d}{8} \right\}$ (3)

Here, $\delta_{\{1, 2,3\}}$ denotes the magnitude factor for losses in different resolution. Practically, $\delta_{\{1, 2,3\}}$ halves with each decrease in resolution, which means $\delta_{2}=\frac{\delta_{1}}{2}$, $\delta_{3}=\frac{\delta_{1}}{4}$, and $\delta_{4}=\frac{\delta_{1}}{8}$. At last, all the weight factors are normalized to 1.

**Supplemental Figure 2.** **The similarity between automatic segmentation and ground truth.**

**
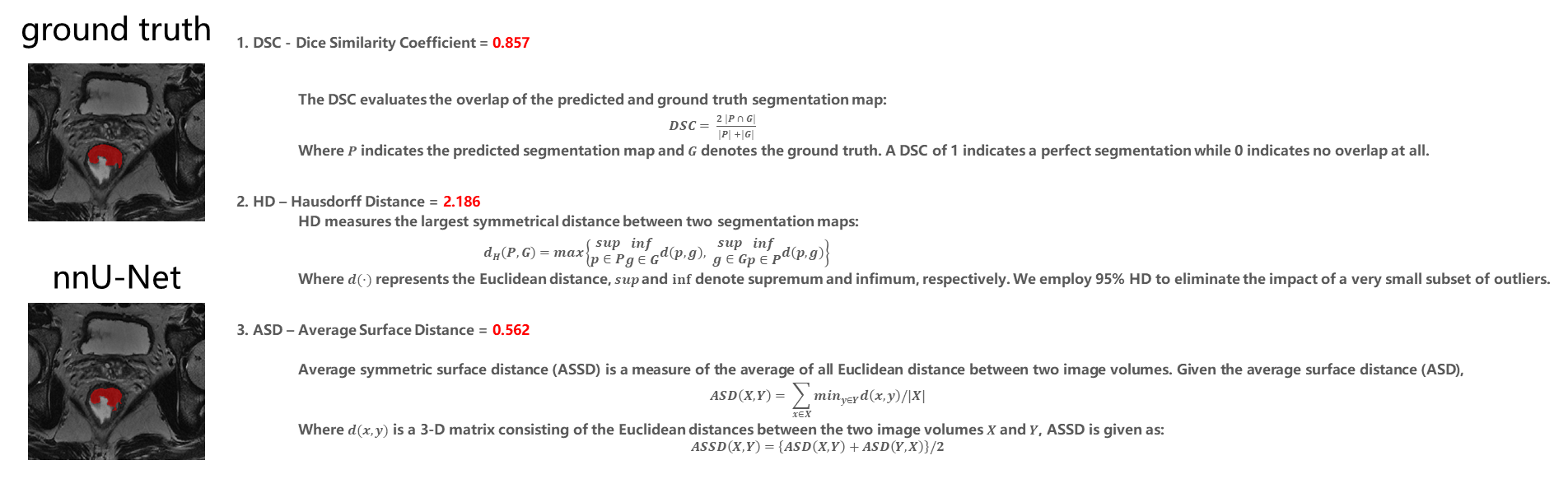
**

The best result of DSC is 1, and the worst result is 0. The higher the score is, the larger the union of the prediction and the ground truth is. The unit of HD95 and ASD is millimeter. The smaller the distance is, the closer the prediction is to the ground truth.

Test set: the mean DSC, HD95, and ASD were 0.857±0.041, 2.186±0.956 mm, and 0.562±0.194 mm, respectively.

External validation set 1: the mean DSC, HD95, and ASD were 0.806±0.120, 4.192±5.477 mm, 1.012±1.269 mm, respectively.

External validation set 2: the mean DSC, HD95, and ASD were 0.844±0.059, 3.046±2.636 mm, 0.690±0.400 mm, respectively.

**Supplemental Figure 3.** **The algorithms on feature selection.**

A


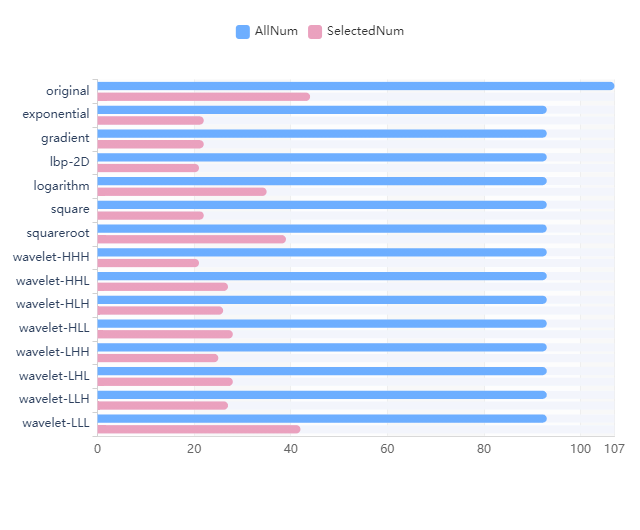


B


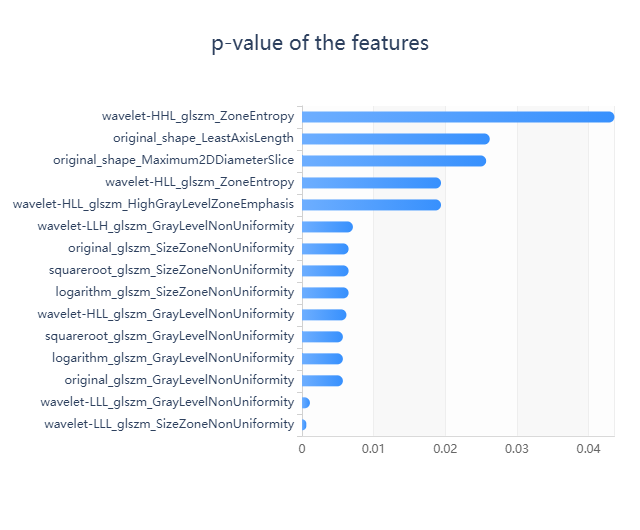


C


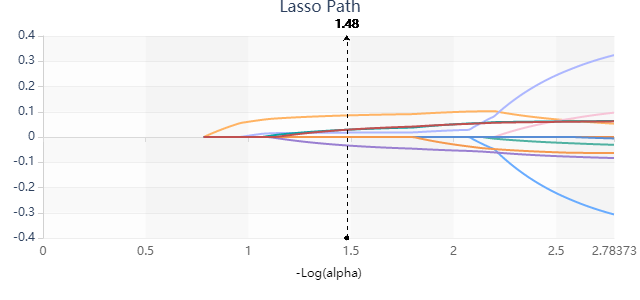


D


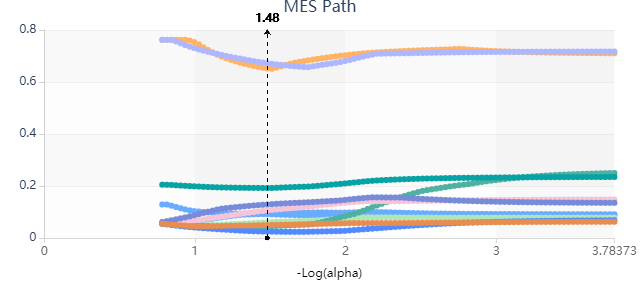


E


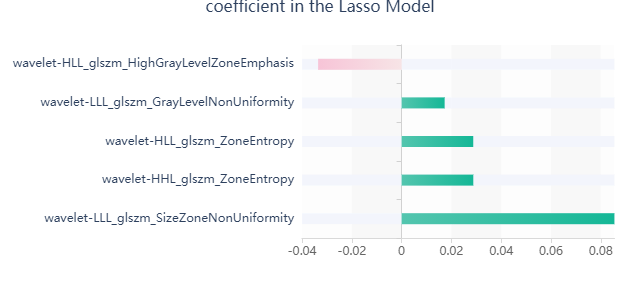


(A) Consistent analysis for feature selection. We used ICC methods to select radiomics features (ICC > 0.8), we selected 429 features from 1409 features.

(B) Select K best on feature select. We used Select K best method to further select radiomics features, we selected 15 features. The Select K Best method, which belongs to a single variable feature selection method, using *p* value to analysis the relationship between the features and the classification results, all the features with *p* value smaller than 0.05 will be used.

(C) LASSO authorism. L1 regularizer was used as the cost function, and the error value of cross validation is 10, and the maximum number of iterations is 1000.

(D) Mean-Squared Error path. The solid vertical lines are deviance ± standard error (SE). The dotted vertical lines are drawn at the optimal values by minimum criteria and 1-SE criteria.

(E) Coefficients in LASSO authorism. Using LASSO model, 5 features which are correspond to the optimal alpha value were selected. Finally, the optimal features with corresponding non-zero coefficients were linearly combined to obtain a Radscore for classification analysis.


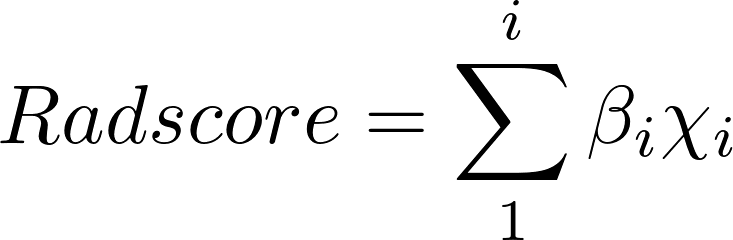


X_i_ refers to the input radiomics features, β_i_ indicates the coefficients.

Radscore＝-0.0379425856* HighGrayLevelZoneEmphasis_GLSZM_wavelet-HLL＋0.0184144997*GrayLevelNonUniformity_GLSZM_wavelet-LLL＋0.0288828837*ZoneEntropy_GLSZM_wavelet-HLL＋0.0290028587*ZoneEntropy_GLSZM_wavelet-HHL＋0.0834653346*SizeZoneNonUniformity_GLSZM_wavelet-LLL

**Supplemental Figure 4.** **The calibration curves of the nomogram.**


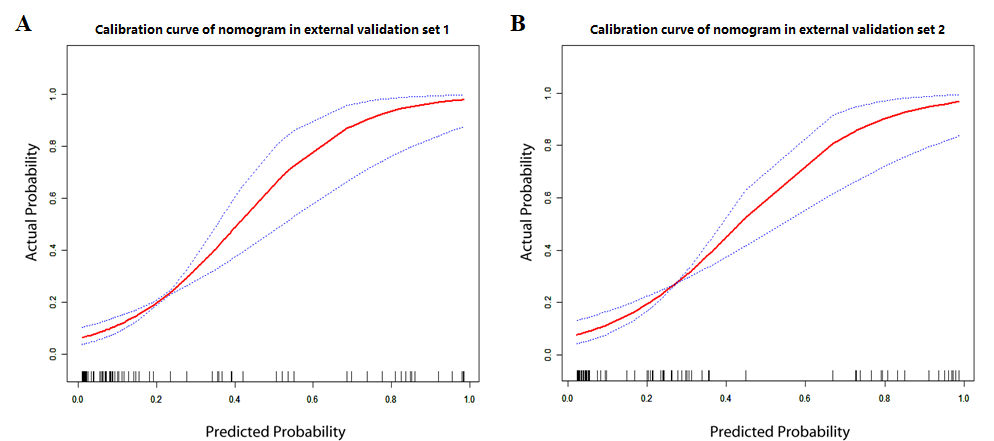


(A) External validation set 1. (B) External validation set 2.
